# A novel germline mutation of the *SFTPA1* gene in familial interstitial pneumonia

**DOI:** 10.1038/s41439-019-0044-z

**Published:** 2019-03-05

**Authors:** Martina Doubková, Kateřina Staňo Kozubík, Lenka Radová, Michaela Pešová, Jakub Trizuljak, Karol Pál, Klára Svobodová, Kamila Réblová, Hana Svozilová, Zuzana Vrzalová, Šárka Pospíšilová, Michael Doubek

**Affiliations:** 1 Department of Pneumology and Phtiseology, University Hospital and Faculty of Medicine, Brno, Czech Republic; 2 Central European Institute of Technology, Masaryk University, Brno, Czech Republic; 3 Department of Internal Medicine, Hematology and Oncology, University Hospital and Faculty of Medicine, Brno, Czech Republic

**Keywords:** Disease genetics, Genetics

## Abstract

Different genes related to alveolar stability have been associated with familial interstitial pneumonia (FIP). Here, we report a novel, rare *SFTPA1* variant in a family with idiopathic interstitial pneumonia (IIP). We performed whole-exome sequencing on germline DNA samples from four members of one family; three of them showed signs of pulmonary fibrosis (idiopathic interstitial pneumonia) with autosomal-dominant inheritance. A heterozygous single nucleotide variant c.532 G > A in the *SFTPA1* gene has been identified. This variant encodes the substitution p.(Val178Met), localized within the carbohydrate recognition domain of surfactant protein A and segregates with the genes causing idiopathic interstitial pneumonia. This rare variant has not been previously reported. We also analyzed the detected sequence variant in the protein structure *in silico*. The replacement of valine by the larger methionine inside the protein may cause a disruption in the protein structure. The c.532 G > A variant was further validated using Sanger sequencing of the amplicons, confirming the diagnosis in all symptomatic family members. Moreover, this variant was also found by Sanger sequencing in one other symptomatic family member and one young asymptomatic family member. The autosomal-dominant inheritance, the family history of IIP, and the evidence of a mutation occurring in part of the *SFTPA1* gene all suggest a novel variant that causes FIP.

## Lung disease: A different type of interstitial pneumonia

Researchers have identified a novel mutation that causes inheritable and ultimately deadly scarring of the tiny air sacs in the lungs, the alveoli. In familial interstitial pneumonia (FIP), alveoli are scarred and stiffened by inflammation, not by bacterial infection as the word ‘pneumonia’ usually indicates. Michael Doubek at University Hospital and Central European Institute of Technology, Brno, Czech Republic and co-workers investigated a suspected case of FIP following the early death of one family member. Sequencing the genomes of other family members revealed that they shared a mutation in a protein that keeps alveoli moist, aiding oxygen absorption. Computer analysis showed that the mutation probably changed the protein’s shape, preventing it from functioning. Identifying mutations that cause FIP will help provide proactive treatment for family members who are at risk but not yet showing symptoms.

## Introduction

Familial interstitial pneumonia (FIP) is defined as idiopathic interstitial pneumonia (IIP) and affects two or more first-degree relatives who have been diagnosed with characteristics of IIP^1^. IIP belongs to a group of interstitial lung diseases (ILDs). ILDs are a heterogeneous group of predominantly chronic diseases characterized by various degrees of inflammation and pulmonary fibrosis at the level of the interstitium, alveolar ducts, alveoli, pulmonary capillaries and respiratory bronchioles^2^.

There is evidence that the development of pulmonary fibrosis is genetically determined, and genetic testing is considered for IIP cases with (1) familial clustering of IIP; (2) IIP associated with other inherited disorders; or (3) when there is significant variability in the development of pulmonary fibrosis among individuals exposed to a dusty environment^3^. Moreover, several phenotypes of fibrotic interstitial lung processes may be present in members of one family. Idiopathic pulmonary fibrosis/usual idiopathic pneumonia (IPF/UIP) is the most frequently observed phenotype in the familial occurrence of IIP^4^. There are no differences in clinical, radiologic, or histological features between familial IPF and nonfamilial IPF cases^5^.

In FIP, many genes may be pathogenic. FIP risk factor genes include telomerase catalytic activity genes (*TERT –* telomerase reverse transcriptase; *TERC –* telomerase RNA component), genes affecting telomerase biogenesis (*DKC1* – dyskerin; PARN *–* polyadenylation-specific ribonuclease deadenylation nuclease; *NAF1 –* nuclear assembly factor 1 ribonucleoprotein) and genes that alter telomeres (*TINF2 –* telomere-interacting factor 2; *RTEL1 –* regulator of telomere-elongation helicase-1). Mutations associated with adult-onset FIP are also rarely found in genes that encode surfactant proteins, such as the heterozygous mutations of surfactant proteins A and C (*SFTPA1, SFTPA2*, and *SFTPC*). Rare biallelic variants in the genes encoding surfactant protein B (*SFTPB*) and branched-chain amino acid aminotransferase (*BCA3*) have also been described. Altogether, mutations in *SFTPC, SFTPA2, TERT*, and *TERC* clarify a maximum of 20% of all FIP cases^6–17^. However, common variants (an allele frequency in the population above 5%) in genes associated with a risk of FIP and IPF have been observed. Most often, the single nucleotide polymorphism (SNP) rs35705950 of the *MUC5B* gene has been strongly associated with both FIP and IPF across multiple different cohorts^18,19^.

Here, we present the FIP case in which we have revealed a novel germline pathogenic variant in the *SFTPA1* gene.

## Materials and Methods

### Subjects

#### The proband

**Fig. 1:**
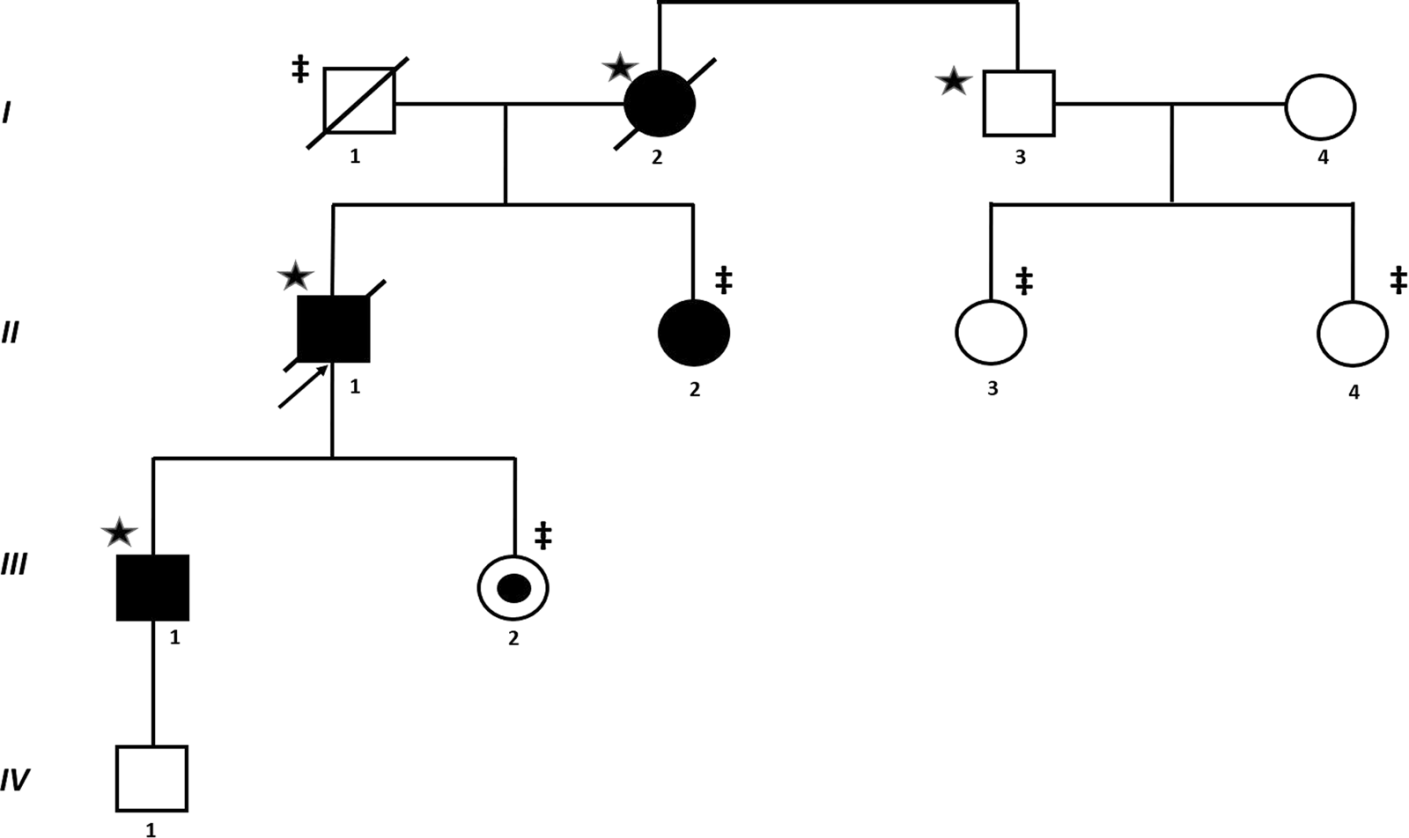
Familial interstitial pneumonia findings. Proband’s (II-1) family pedigree shows the segregation of pulmonary fibrosis (idiopathic interstitial pneumonia). Samples analyzed by exome sequencing and subsequent Sanger sequencing are marked with an asterisk (*); the samples analyzed by Sanger sequencing only are marked with a double-dagger (‡)

**Fig. 2:**
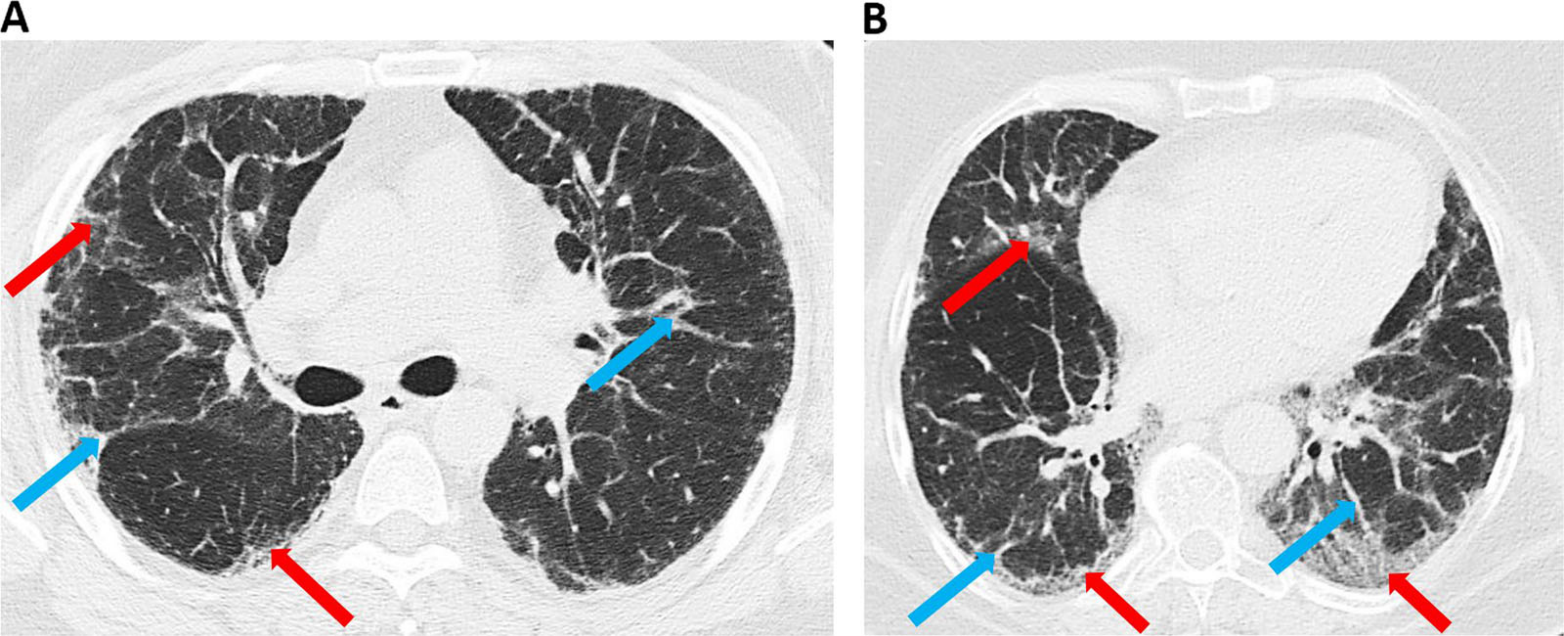
Chest high-resolution computed tomography findigs. Chest HRCTs (high-resolution computed tomography; transverse plane) of the proband (II-1) (**a**) and the proband´s mother (I-2) (**b**). Inter- and intralobular septal thickening and irregular reticular lines (blue arrows), ground glass opacities arrows, and crazy paving and traction bronchiectasis without honeycombing (red arrows) are shown

Figure 1 (*II-1*) was a male nonsmoker who was referred to the Department of Pneumology and Phtiseology, University Hospital, Brno, Czech Republic because of IIP at the age of 46 years. The diagnosis of IIP was based on standard criteria^2^. The proband presented with dyspnea and a dry cough. Lung function testing showed a restrictive syndrome with a mild reduction in diffuse lung capacity. High-resolution computed tomography (HRCT) of the patient’s chest showed interlobular septal thickening and ground glass opacities, especially in the lower lung areas (Fig. 2a). Video-assisted thoracoscopic (VATS) lung biopsy revealed pulmonary fibrosis with a nonspecific interstitial pneumonia pattern. He died of respiratory failure due to acute exacerbation of pulmonary fibrosis 11 years after diagnosis.

A detailed family history revealed that several of the patient’s family members had pulmonary fibrosis or interstitial lung involvement. The proband´s mother (*I-2*), a nonsmoker, died of respiratory failure at the age of 73 years. She was diagnosed with IIP seven years ago. HRCT of the thorax initially showed predominant ground glass opacities, inter- and intralobular septal thickening and bronchiectasis that did not correspond with the possible, typical interstitial pneumonia (Fig. 2b). However, a VATS lung biopsy and histological examination revealed a pattern of typical interstitial pneumonia. The clinical course gradually deteriorated over time despite therapy.

### The proband´s sister

(II-2), a nonsmoker with digital clubbing, developed dyspnea at the age of 44 years. An HRCT of her thorax showed ventral and dorsobasal subpleural interlobular septal thickening.

### The proband´s son

(III-1), a nonsmoker, had suffered from shortness of breath during exercise since the age of 25. He also developed digital clubbing. An HRCT of his thorax revealed discrete nonspecific ground glass opacities in the upper and lower right lobes.

### The proband´s daughter

(*III-2*), a nonsmoker, was asymptomatic.

All results of the clinical and radiology examinations, functional tests and treatment of family members with pulmonary symptoms are summarized in Supplemental Table S1.

The pedigree analysis (Fig. 1) indicates an autosomal-dominant mode of inheritance.

FIP was therefore suspected, and diagnostics were extended by genetic testing of the family members, who gave written informed consent according to the Declaration of Helsinki.

### Mutational screening

Blood samples from nine family members were collected and processed for genomic DNA isolation using the MagCore® Genomic DNA Whole Blood Kit (RBC Bioscience, USA). We performed whole-exome sequencing (WES) on samples from four family members (I-2, I-3, II-1, and III-1). Whole-exome libraries were prepared using the Kapa Hyper Prep Kit (Roche, USA) according to the protocol for NimbleGen SeqCap EZ Exome v3 (Roche, USA). Paired-end 2 × 75 bp sequencing was performed on an Illumina NextSeq 500 sequencer (Illumina Inc., USA). The raw sequencing reads were aligned to the GRCh37 human reference genome using the BWA mem algorithm, version 0.7.15. PCR duplicates were identified with the MarkDuplicates tool from Picard version 2.9.2. GATK HaplotypeCaller, version 3.7, was used to detect germline single nucleotide variants (SNV) and indels. Obtained variants/indels have been annotated using Annovar program version (2018Apr16).

On the basis of the current knowledge, we have chosen 30 candidate genes previously associated with *IPF*: *TERC*, *TERT*, *SFTPC*, *SFTPA1*, *SFTPA2*, *MUC5B*, *MUC5C*, *RTEL1*, *PARN*, *ABCA3*, *DKC1*, *TINF2*, *IL1RN*, *IL8*, *FAM13A*, *TLR3*, *HLA- DRB1*, *HLA- DQB1*, *DSP*, *OBFC1*, *MUC2*, *TOLLIP*, *ATP11A*, *MDGA2*, *MAPT*, *SPPL2C*, *DPP9*, *TGFB1*, *NAF1*, and *OBFC1*^7–17^. We then looked more deeply into the exonic variants of these genes.

## Results

The analysis revealed a novel variation c.532 G > A in exon 6 of the *SFPTA1* gene (reference sequence: NM_005411.4), located within the carbohydrate recognition domain of surfactant protein A. The variant was found to be heterozygous in three affected family members (I-2, II-1, III-1) with IIP but was absent in the healthy individual I-3 (Fig. 1). The coverage range of c.532 G > A was 66–126 in all affected samples, and the variant allele frequency range was 41.41%-59.09%. This identified variant (c.532 G > A) has not been described previously and is absent in the ExAC, 1000 genomes, ESP (exome sequencing projects) 6500, KAVIAR, gnomAD, and HGMD databases^20,21^.

**Fig. 3:**
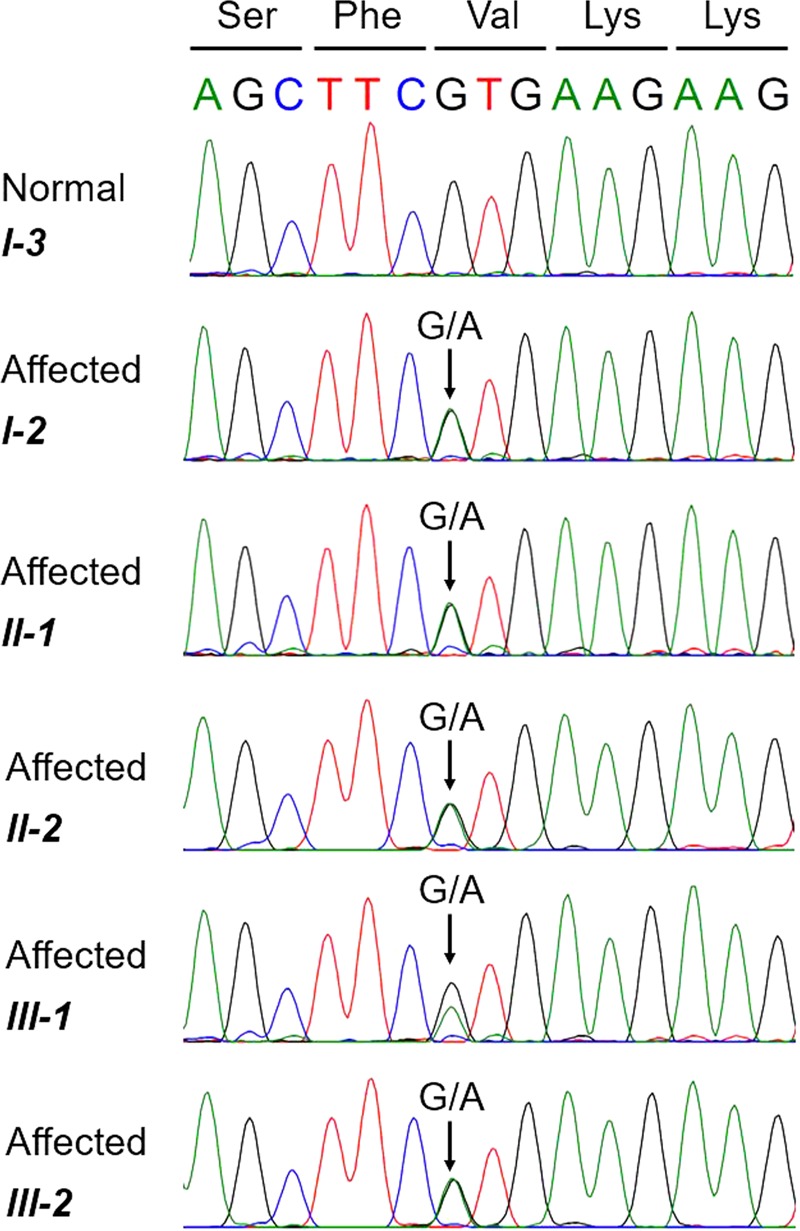
Familial interstitial pneumonia findings. Chromatograms depicting the capillary sequencing results of the c.532 G > A; p.(Val178Met) sequence variant in all affected individuals and in one non-affected family member. Coded amino acids are also shown

The c.532 G > A variant was further validated using PCR and Sanger sequencing of the amplicons, confirming the diagnosis in all affected family members (I-2, II-1, and III-1). Due to the similarity of the *SFTPA1* and *SFTPA2* genes, we designed a specific primer set corresponding to the differences in the nucleotide sequence (forward primer: 5´-TGGTCAGTGGCCTGACCC-3´ and reverse primer: 5´-AGAGTCAGGGCCCATCAGA-3´). PCR was performed with an annealing temperature of 60 °C using Q5 High-Fidelity DNA Polymerase (New England Biolabs Inc., England) according to the manufacturer´s protocol. PCR products were purified using a Qiaquick PCR purification kit (QIAGEN, Germany). Capillary sequencing was performed using BigDye-terminator chemistry on a 3500 Genetic Analyzer (Applied Biosystems, USA). The analysis showed that the variant c.532 G > A was heterozygous in the I-2, II-1, II-2 and III-1 family members (Fig. 3). Subsequently, PCR and Sanger sequencing of c.532 G > A was performed in the other family members (I-1, II-2, II-3, II-4, and III-2). Moreover, the c.532 G > A heterozygous variant was found in two additional family members: II-2 with disease symptoms and III-2, the young but still asymptomatic family member.

### In silico analysis

Currently, there is no evidence for any known functional impacts of the c.532 G > A; p.Val178Met variant in the *SFPTA1* gene, which encodes the missense substitution valine to methionine in the α-helix (the carbohydrate recognition domain of surfactant protein A1). Valine178 is a highly conserved amino acid and is found in up to 11 frog species. We performed an assessment of the severity of the identified variant using *in silico* prediction analysis: the Sorting Intolerant from Tolerant (SIFT), Polymorphism Phenotyping v2 (Poly-Phen-2), MutationTaster, Align GVGD and CADD_phred programs (http://sift.jcvi.org/www/SIFT_seq_submit2.html; http://genetics.bwh.harvard.edu/pph/; http://www.mutationtaster.org/; http://agvgd.hci.utah.edu/agvgd_input.php)^22^. According to SIFT, this variant with a score of 0.01 was “deleterious” (the score of predicted damage was < 0.05). The Poly-Phen-2 program evaluated p.Val178Met as “probably damaging” with a score of 1,0 (a maximum score). On the other hand, MutationTaster predicted this variant as a “polymorphism” with a p-value of 1 (score of predicted damage is < 0.09), and the Align GVGD (Grantham Variation and Grantham Deviation) defined “the lowest risk” for the prediction of missense substitutions with the grade C0 (C0 is the first of a total of seven grades). However, the scaled CADD_phred score was calculated as 16.72. This result of scaled transformation was assigned to the top 1% of single nucleotide variants and may predict a potentially pathogenic variant.

We therefore analyzed the c.532 G > A; p.Val178Met variant effects via protein structure modeling. Currently, there is no available crystal structure of this human protein, so we used the homolog protein structure of porcine surfactant protein D neck and carbohydrate recognition domains complexed with mannose (PDB code 4DN8), which shows 43% sequence homology with the human protein. In the homology model, valine178 is positioned in the α-helix, which is buried inside the protein. The replacement of this valine (its volume is 140.0 Å^3^)^23^ by larger methionine (its volume is 162.9 Å^3^) will probably cause a disruption of the protein structure (supplemental Figure S1).

## Discussion

Unfortunately, we do not have accurate FIP prevalence and incidence data. However, some studies estimate that the incidence of FIP is approximately 2-10% of all IIP cases^1,3,8,12^. The inheritance of FIP is most commonly autosomal-dominant with incomplete penetrance^4,5,19,24–26^. Autosomal-recessive types of inheritance have rarely been described^27^. Our case shows signs of an autosomal-dominant inheritance.

Both familial and sporadic cases of pulmonary fibrosis are not clinically or histologically recognizable, although familial cases can be diagnosed in younger individuals^3^. Rosas et al.^28^ determined the clinical, radiographic, and histological features of asymptomatic ILDs in the relatives of patients with FIP. Histopathological findings in patients with FIP are heterogeneous^29^. Steele et al.^4^ described several histopathological subtypes of IIP found in members of one family. Our case also shows different histological, clinical and radiological findings in affected individuals.

FIP is caused mainly by rare pathogenic gene variants (with an allele frequency in the population of less than 0.1%)^30^. Familial studies have found FIP-associated genes influencing alveolar stability: *SFTPA1*^15^, *SFTPA2*^14^, and *SFTPC*;^30–32^ *ABCA3* (ATP-binding cassette - type 3);^32^ genes related to telomerases, including *TERT*^29^, and *TERC*;^6,29^ *DKC1* (dyskeratin);^8,29^ *TINF2*;^7,9,29^ or *RTEL1*^10,11,29,33^. On the other hand, common variants are also observed in FIP; most often, the variant rs35705950 is located in the promoter region of the *MUC5B* gene^29,34^.

Surfactant proteins (SPs) are divided into hydrophilic (SP-A and SP-D) and hydrophobic (SP-B and SP-C) categories. SP-A, SP-B, SP-C and SP-D are synthesized by type II alveolar epithelial cells. SP-A and SP-D play roles in lung defense, and SP-B and SP-C ensure proper surfactant function^35,36^. The main protein of pulmonary surfactant is SP-A^37^.

The two closely related genes, *SFTPA1* and *SFTPA2*, are located near *SFTPD* on chromosome 10q22.3 and encode SP-A. The nucleotide sequence of the *SFTPA1* gene is 70% identical to that of *SFTPA2*. The pathogenic variants of *SFTPA1* and *SFTPA2* can cause FIP and pulmonary adenocarcinoma. All pathogenic heterozygous mutations in the *SFTPA1* and *SFTPA2* genes are missense and lead to a decreased secretion of mature protein by alveolar cells^15,38–40^.

In our case, the novel single nucleotide variant c.532 G > A was detected in the *SFTPA1* gene and encodes a p.Val178Met substitution.

To date, 5 missense/nonsense *SFTPA1* variants have been identified (https://portal.biobase-international.com/hgmd/pro/gene.php?gene = SFTPA1; 09 AUG 2018)^15,20,41^. Nathan et al. described in detail a molecular defect in *SFTPA1* in FIP^15^.

Unfortunately, there is no consensus to determine which IPF patient could benefit from genetic testing. The international guidelines for IPF did not recommend genetic testing^42^, and genetic testing is not even mentioned in the guidelines^43^. On the other hand, the international guidelines for IIP published in 2013 propose searching for genetic abnormalities in patients with FIP^44^. As proposed by Kropski et al.^45^, genetic testing is not recommended for every ILD patient. Genetic counseling should be offered to patients with FIP, to patients with IIP in the context of rare inherited disorders, to individuals with disease onset before the age of 18 years, or to patients with significant variability in the development of pulmonary fibrosis after exposure to a dusty environment^3,45^. For asymptomatic patients (similar to our patient III-2), a chest HRCT is recommended at age 40 or 10 years before the age of disease onset in the proband. If signs of ILD are not present, an HRCT scan should be repeated every 5 years^35^.

In conclusion, we have described a novel *SFTPA1* heterozygous variant in family members with IIP. Such findings are of the utmost importance, and an early FIP diagnosis for the patient’s family members will allow for effective therapies, including early lung transplantation or treatment, following clinical trials.

## Supplementary information

Supplemental Figure legend and supplemental Table

Supplemental figure
