# Supplementary material for: A novel germline mutation of the SFTPA1 gene in familial interstitial pneumonia
Source: Hum Genome Var. 2019 Mar 5;6:12. doi: 10.1038/s41439-019-0044-z (PMC6399245; doi:10.1038/s41439-019-0044-z)
Supplement: Supplementary file 1 — Supplemental Figure legend and supplemental Table [file 41439_2019_44_MOESM1_ESM.docx]

**A novel germline mutation of *SFTPA1* gene in familial interstitial pneumonia**

Martina Doubková^1‡^, Kateřina Staňo Kozubík^2,3‡^, Lenka Radová^2^, Michaela Pešová^2^, Jakub Trizuljak^2,3^, Karol Pál^2^, Klára Svobodová^2^, Kamila Réblová^2^, Hana Svozilová^2,3^, Zuzana Vrzalová^2,3^, Šárka Pospíšilová^2,3^, and Michael Doubek^2,3^

^1^ Department of Pneumology and Phtiseology, University Hospital and Faculty of Medicine, Brno, Czech Republic

^2^ Central European Institute of Technology, Masaryk University, Brno, Czech Republic

^3^ Department of Internal Medicine, Hematology and Oncology, University Hospital and Faculty of Medicine, Brno, Czech Republic
‡ These authors contributed equally to this work

**Supplemental Figure**

**Figure S1.** Detailed view on 3D structure of homologous model of human SFTPA1 protein built based on 4DN8 template structure. Valine178 (in green) is positioned in the α-helix, which is buried inside the protein. Replacement of this valine by larger methionine can cause disruption of the protein structure.

**Supplemental Table**

**Table S1.** Results of clinical and radiology examination and functional tests of affected family members. HRCT – high-resolution computed tomography.

|  | **Patients** | | |  |
| --- | --- | --- | --- | --- |
|  | ***I-2*** | ***II-1* (proband)** | ***II-2*** | ***III-1*** |
| **Age at diagnosis (years)** | 66 | 46 | 44 | 25 |
| **Smoking** | Ex-smoker | Non-smoker | Non-smoker | Non-smoker |
| **Symptoms** | Dyspnea and cough | Dyspnea and cough | Dyspnea in exercises | Dyspnea in exercises |
| **Digital Clubbing** | No | No | Yes | Yes |
| **Auscultation** | Crepitations on lung bases | Crepitations on lung bases | Discrete crepitations on lung bases | Normal |
| **Bronchoalveolar lavage** | Neutrophilic and eosinophilic alveolitis | Normal finding | *Not performed* | *Not performed* |
| **Functional tests** | Mild restrictive impairment, mild decreased diffuse lung capacity | Mild restrictive impairment, mild decreased diffuse lung capacity | Normal ventilation, including spiroergometry | Normal ventilation, including spiroergometry |
| **Chest X-ray** | Reticulations on lung bases | Reticulations on lung bases | Normal finding | Normal finding |
| **HRCT** | Ground glass opacities inter– and intralobular thickening, bronchiectasis in the lower and middle lung zones; bilateral | Interlobular septal thickening and ground glass opacities in the lower lung zones | Ventral and dorsobasal subpleural interlobular septal thickening; mild impairment | Discrete nonspecific ground glass opacities in the upper and lower right lobes |
| **Histology** | Usual interstitial pneumonia | Nonspecific interstitial pneumonia | *Not performed* | *Not performed* |
