# Supplementary figures and images for: A novel germline mutation of the SFTPA1 gene in familial interstitial pneumonia
Source: Hum Genome Var. 2019 Mar 5;6:12. doi: 10.1038/s41439-019-0044-z (PMC6399245; doi:10.1038/s41439-019-0044-z)

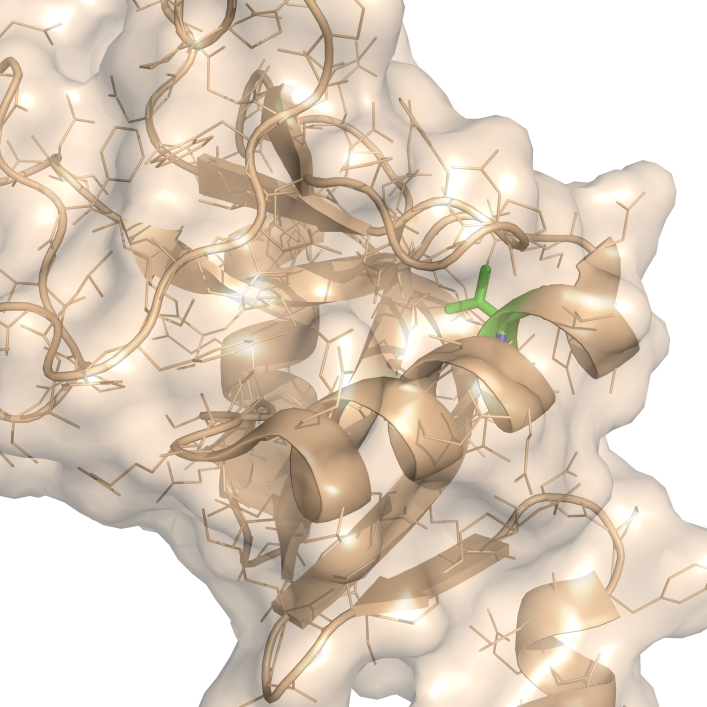

Supplement: Supplementary file 2 — Supplemental figure [file 41439_2019_44_MOESM2_ESM.tif]
